# Impact of addictive behaviors on productivity at work among employees working on an onshore oil field

**DOI:** 10.1192/j.eurpsy.2021.1504

**Published:** 2021-08-13

**Authors:** N. Rmadi, N. Kotti, R. Masmoudi, F. Dhouib, K. Jmal Hammami, M. Larbi Masmoudi, J. Masmoudi, M. Hajjeji

**Affiliations:** 1 Department Of Occupational Medicine, HEDI CHAKER hospital, SFAX, Tunisia; 2 Psychiatrie “a” Department, Hedi Chaker Hospital University -Sfax - Tunisia, sfax, Tunisia

**Keywords:** addictive behaviors, onshore oil workers

## Abstract

**Introduction:**

Addictive behaviors on the workplace are a real public health problem because of its consequences not only on workers but also on productivity at work.

**Objectives:**

To explore the relationship between addictive behaviors and productivity at work among employees of a Tunisian oil rig.

**Methods:**

A cross-sectional study was conducted in the first half of 2018. The assessment of work productivity was done using the validated WPAI-GH questionnaire. Smoking dependence was assessed via the Fagerström score and alcohol abuse by the FACE questionnaire.

**Results:**

It was 94 employees working in an onshore oil field with an average age of 41.1 years. Average job seniority was 14.3 years. Active smoking was noted in 34.7% of cases. Alcohol consumption was noted in 19.1% of cases. In the 7 days preceding the survey, the average percentage of absenteeism was 3.64 ± 21.7% and the presenteeism was 17.66 ± 25.58%. The average decline in productivity was 14.8 ± 43.7% and the average decline in daily activities was 20.21 ± 31.45%. These parameters were not correlated with smoking and alcoholism.

**Conclusions:**

Addictive behaviors in the workplace still a denied reality.Increasing awareness and clarifying expectations can be a good first step in order to ameliorate employee functioning and decrease productivity problems.

